# Arrayed Magnetic Accelerator Synergistically Enhances Orthodontic Tooth Movement via Microtubule Assembly‐Mediated Periodontal Remodeling

**DOI:** 10.1002/advs.77447

**Published:** 2026-10-04

**Authors:** Yiling Lou, Jiwei Sun, Peng Chen, Caihong Mao, Junyuan Zhang, Haoqi Lei, Xiaoqing Han, Yaxuan Zhang, Yuheng Jiang, Yulin Jia, Bin Su, Lili Chen

**Affiliations:** ^1^ Department of Stomatology Union Hospital Tongji Medical College Huazhong University of Science and Technology Wuhan China; ^2^ School of Stomatology Tongji Medical College Huazhong University of Science and Technology Wuhan China; ^3^ School of Materials Science and Engineering Huazhong University of Science and Technology Wuhan China

**Keywords:** accelerated orthodontic tooth movement technology, orthodontics, static magnetic field, tooth movement efficiency

## Abstract

Mechanical orthodontic treatment is regarded as a classical strategy for directional tooth movement in confront of malocclusion. However, prolonged remodeling period and adverse complication risks, which are mainly ascribed to inadequate sensation of bio‐mechanical cues, predominantly restrict orthodontic clinical outcomes. This study demonstrates that construction of biophysical static magnetic fields (SMFs) is highly involved in accelerated tooth movement and bone remodeling in manners dependent on amplification of mechanical sensing. The arrayed magnetic orthodontic accelerator (AMOA) with precisely controllable SMFs was fabricated by 3D printing. Exposure to SMFs can enhance production of pro‐inflammatory cytokines from periodontal ligament cells (PDLCs) through mechanical sensitization to facilitate polarization of M1‐like macrophages and differentiation of osteoclasts for improved bone remodeling. Pre‐clinical exploitation clarifies that AMOA application dramatically facilitated alveolar bone remodeling and osteoclastic activity in the compression side of orthodontic tooth model. In summary, our results suggest that SMFs‐upregulated sensing of biological force in PDLCs participates in orthodontic tissue remodeling and plays a pivotal role in acceleration of tooth movement. This study sheds light on the therapeutic transformation of AMOA to improve clinical outcomes of classical orthodontic treatment.

## Graphical abstract

Mechanical orthodontic treatment for malocclusion is limited by slow remodeling and risks due to poor biomechanical sensing. This study shows that static magnetic fields (SMFs) enhance mechanical sensation in periodontal ligament cells, promoting inflammation and osteoclast activity. A 3D‐printed magnetic orthodontic accelerator (AMOA) accelerates tooth movement and alveolar bone remodeling, offering improved clinical outcomes.

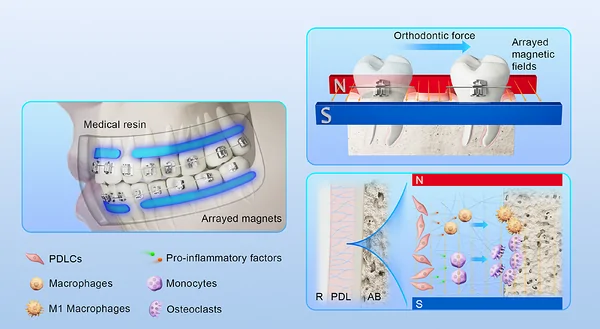

## 1 Introduction

Malocclusion nowadays acts as a universal oral health issue with a remarkable prevalence up to almost 60% worldwide [1]. Malocclusion‐associated disorders would result in series of poor clinical outcomes including dentofacial and stomatognathic dysfunctions [2, 3]. Application of mechanical force is the classical scheme for orthodontic tooth movement (OTM) via a cyclic alternating mechanical stimulation. However, single mechanical regulation frequently leads to prolonged treatment period, adverse complications and fails to achieve efficient and fast OTM process [4, 5]. Current methods for acceleration of OTM mainly focus on operation treatment and systemic drug administration [6]. Nevertheless, surgical operations would cause evident tissue damage which might delay remodeling of periodontal regions [7]. Systemic medication is limited due to its narrow indication scope and debatable clinical effect [8]. In recent years, biological strategies using growth factors (e.g., BMPs, PGE_2_) and nanoscale vesicles (including exosomes, stem cell‑derived intracellular vesicles, and inosomes) have emerged as potential approaches to modulate OTM and prevent post‑treatment relapse [9, 10, 11]. For instance, mesenchymal stem cell‑derived exosomes regulate periodontal bone remodeling by modulating osteoclast activity [9]; dental pulp stem cell‑derived intracellular vesicles inhibit osteoclast‑mediated relapse via PI3K/Akt/NF‑κB signaling [10]; and inosome‑based platforms offer a cell‑free delivery system for orthodontic retention [11]. However, these biological methods often require repeated local injections, face challenges in controlled release, and have not yet achieved clinical translation. In contrast, magnetic appliances provide a non‑invasive, patient‑friendly physical modality that can be applied continuously without exogenous biological agents. Therefore, clinical mechanical orthodontics and traditional auxiliary methods are still confronted with challenges in improving orthodontic efficiency and shortening treatment course.

Alveolar bone is mechanosensitive locus which could be remodeled by exertion of functional mechanical loading. OTM is characterized by force‐mediated local aseptic periodontal inflammation. During this process, periodontal ligament cells (PDLCs), main participants located within periodontal ligament, play a central role in initiation of tissue remodeling as they can directly sense mechanical signals for subsequent reconstruction of necessary microenvironment [12]. In particular, PDLC‐induced cascade regulation on macrophage‐derived cellular functionalization has been considered to make huge contributions to efficient bone remodeling. Upon orthodontic force application, PDLCs would express elevated levels of pro‐inflammatory cytokines including interferon (IFN)‐γ, interleukin 6, C‐C motif chemokines (CCL2 and CCL5) and so on [13, 14, 15]. Therefore, communications between PDLCs and macrophages would result in 2 outcomes: 1. Differentiation into osteoclasts for osteoclastic activities, which is main effector for bone remodeling. 2. Polarization into M1‐like macrophages for reinforcement of local inflammation, which is vital for local microenvironment [16, 17]. Based on this theory, stronger orthodontic force will lead to faster bone remodeling and tooth movement. However, incidence of severe adverse complications, including tooth loosening, root resorption and bone fenestration, will take place under constant high‐level mechanical loading [18, 19]. Thus, how to achieve efficient clinical OTM using light force application in combination with auxiliary method still remains as an unsolved challenge.

SMFs used to manifest a characteristic of mystery due to its non‐contact manner and controversial view in biological effect. With the development of experimental techniques during recent years, reliable observations have been obtained to confirm the potential positive effect of SMFs in regulation of multiple biological processes [20, 21, 22]. Among them, role of SMFs in bone‐associated diseases, including osteoporosis, bone loss and bone fracture, has been specifically emphasized, along with generation of medical apparatus based on SMFs effect [23, 24]. However, less attention has been attracted to effect of SMFs in alveolar bone remodeling during orthodontic treatment. It has been previously reported that experimental animals were fed within a customized cage equipped by magnets while receiving OTM treatment, and revealed that systemic exposure to SMFs might enhance the speed of OTM, but this study fails to prove whether precise local application of SMFs into periodontal regions could promote tooth movement [25]. Furthermore, inherent mechanism of SMFs‐assisted OTM still holds unclear. Evidence has suggested that single SMFs could promote proliferation, migration and differentiation of PDLCs [26], while the synergistic mechanism of orthodontic loading and SMFs in regulation of PDLCs‐mediated cascade responses in periodontal microenvironment has not been elucidated.

Our study successfully realizes precise formation of local SMFs via utilizing 3D printing approaches to fabricate an arrayed magnetic orthodontic accelerator (AMOA). Maxwell numerical simulation was performed before fabrication to precisely control distribution and strength of local SMFs. It is further demonstrated that local application of 30mT SMFs via AMOA can significantly promote OTM process in manners dependent on amplification of mechano‐transduction of orthodontic force to PDLCs, which can facilitate M1‐like polarization and osteoclastic differentiation for construction of a pro‐remodeling microenvironment (Scheme 1). This enhanced mechano‐biological signal transduction is demonstrated to be achieved through the promotion of microtubule‐integrin assembly by the static magnetic field (Scheme 2). These results highlight the significance of SMFs in regulating periodontal osteoclastic cascades, supporting the potential clinical utilization of AMOA for achievement of light‐force orthodontics.

**SCHEME 1:**
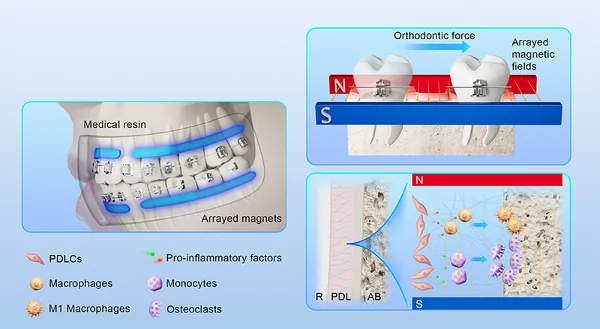
Schematic illustration of orthodontic tooth movement acceleration by AMOA. AMOA facilitates the transduction of mechanical force signals to biological processes under orthodontic force, thereby enhancing inflammatory activity and periodontal remodeling within the periodontal microenvironment, ultimately leading to accelerated orthodontic tooth movement. R: tooth root, PDL: periodontal ligament, AB: alveolar bone.

**SCHEME 2:**
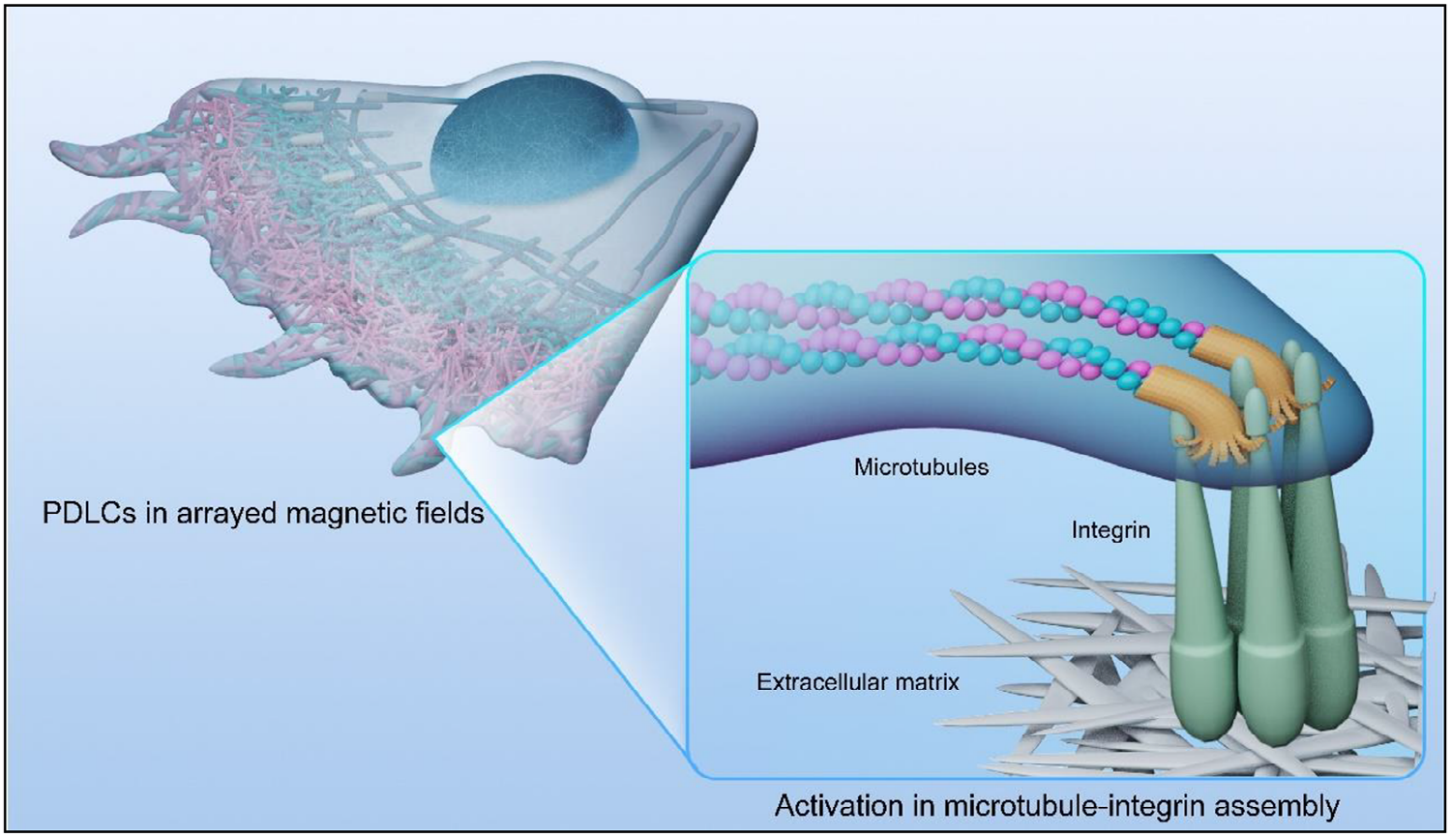
Schematic illustration of the mechanism by which SMFs enhance the perception of mechanical stress signals in periodontal ligament cells. SMFs promote the assembly of microtubule‐integrin complexes, thereby enhancing the downstream signaling cascade initiated by the focal adhesion complex in response to mechanical stimuli. This ultimately results in the reinforcement of mechanotransduction from mechanical signals to biological responses.

## 2 Results

### 2.1 Design and Fabrication of AMOA Applied in Animal Orthodontic Model by Utilizing Customized 3D Printing Techniques

Design and fabrication of matched magnetic device acts as the first step for unveiling the role of local magnetic field in orthodontic tooth movement. To accurately adapt to the size, structure and anatomic morphology of oral cavity, micro‐CT scanning was initially carried out to reconstruct digital oral structure of rats, which facilitated the customized design of experimental AMOA using SLA 3D printing methods (Figure 1a). Design of AMOA was exhibited in Figure 1b, as the device was exactly loaded within periodontal regions. The first molar under the induction of orthodontic force, was surrounded by magnetic modules on the buccal and lingual sides to ensure the exact local application of SMFs for orthodontic periodontal regions. Accordingly, the devices were successfully fabricated (Figure 1c). The most crucial part for experimental AMOAs lays on precise control of SMFs distribution. To achieve this purpose, finite element simulating analysis was performed to form SLA molding strategy. Simulating results indicated that by adjusting molding formulas, AMOAs with low‐level and high‐level SMFs might be manufactured (Figure 1d). Subsequently, different types of AMOAs were developed based on simulating directions, and detection of actual SMFs distribution indicated that accurate control of local SMFs can be successfully achieved (Figure 1e,f), which might contribute to our exploration of biological effects and optimum parameters for SMFs‐mediated orthodontic acceleration. Then, efforts were made to construct animal model using AMOAs. In detail, orthodontic force at 50g was first loaded to the first molar of rats (Figure 1g), followed by daily application of SMFs stimulation by orally using AMOAs for 30 mins (Figure 1h). This exposure duration was selected based on the randomized controlled trial by Alqaisi [27], which demonstrated that 1 h of daily SMFs exposure significantly accelerated orthodontic tooth movement. Considering patient compliance and the fact that the peak magnetic field intensity is the key parameter for mechanotransduction, we adopted a clinically pragmatic 30‑min daily protocol that balances therapeutic efficacy with practical wearability. With the help of gaseous anesthesia, experimental rats could stay calm during the whole period to ensure stable and accurate placement of AMOAs within orthodontic periodontal regions (Figure 1i,j). The whole period safely continues for 4 weeks, evidenced by normal structures of livers and kidneys from experimental rats (Figure S1b,c). In summary, experimental AMOA devices with precise SMFs distribution were successfully fabricated using simulating calculation and SLA printing methods, and animal experimental model was established to study the effects of local SMFs in orthodontic treatment in advance.

**FIGURE 1:**
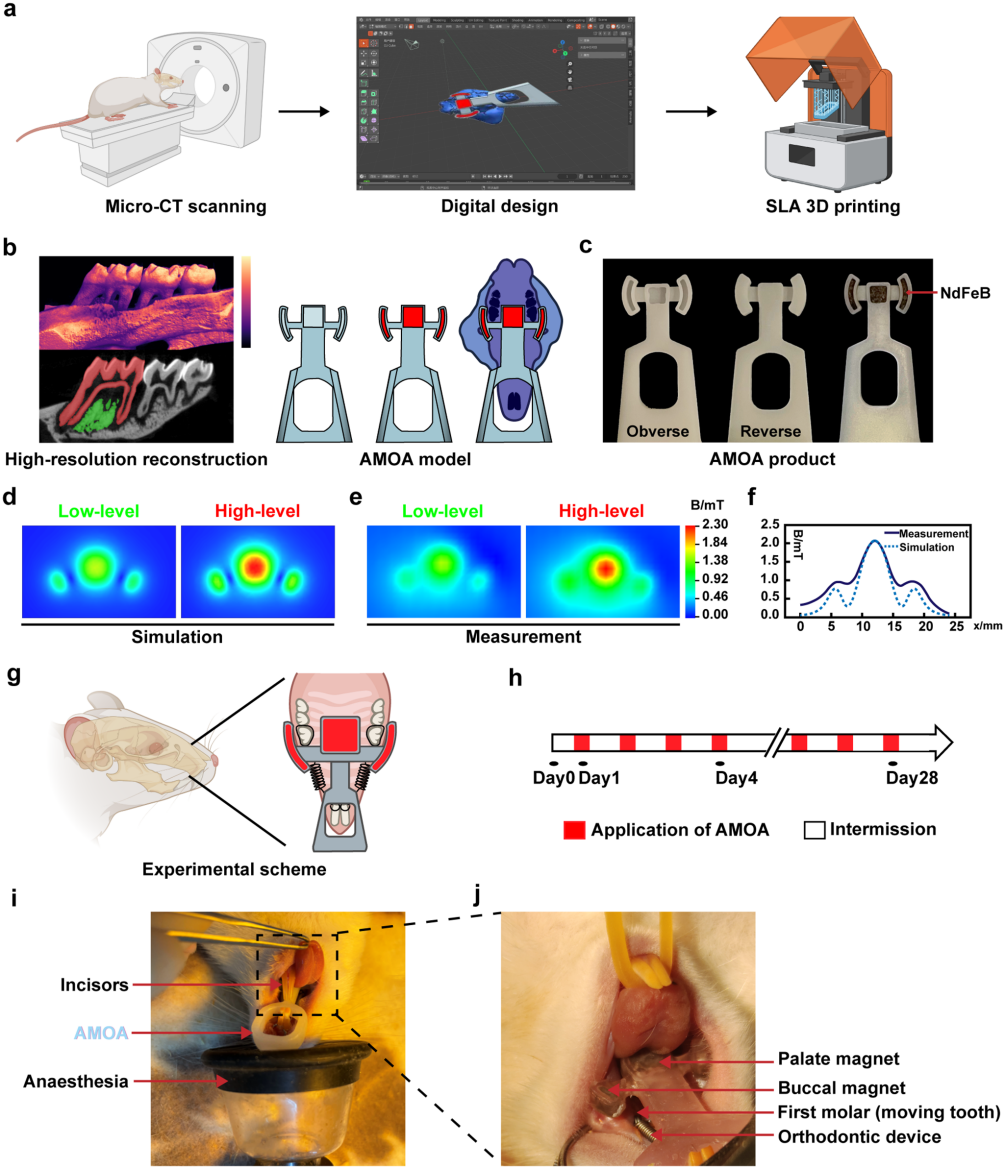
Design of AMOA and application in animal experiments. (a) Micro‐CT scanning and 3D reconstruction of rat maxilla were used for the subsequent AMOA model design and SLA 3D printing. (b) CT image reconstruction of the rat maxilla and the model design of AMOA. (c) The finished product of AMOA, then filled with NdFeB magnets. (d) Expected magnetic field strength under simulation. (e) The magnetic field strength obtained from the actual measurement. (f) Image of the fitting function for the relationship between magnetic field strength and horizontal distance (mm). (g) Schematic representation of the animal experimental setup. (h) The timeline of the animal experiment, in which the experimental group was exposed to SMFs for 4 h per day for 28 days. (i)(j) Actual scenes of the animal experimental setup.

### 2.2 SMFs Accelerate OTM Process and Facilitated Alveolar Bone Remodeling

To estimate the effects of SMFs on orthodontic tooth movement, micro‐CT scanning and analysis was conducted on the seventh, fourteenth, and twenty‐eighth days after orthodontic force application. The results showed that AMOA‐applied group exhibited augmented moving distance within the same time interval compared with those under sole orthodontic force influence (Figure 2a,b). As alveolar bone remodeling acts as the main reason for tooth movement, further analysis also revealed that bone volume fraction, bone density and thickness of bone trabecula were significantly decreased, altogether suggesting that SMFs promoted the reconstruction of alveolar bone under orthodontic force (Figure 2c). Subsequently, we observed the changes in the periodontal ligament (PDL) space. After force application, the PDL on the pressure side in both groups was compressed and narrowed, and the PDL width decreased on the seventh day. However, the PDL width in the Force + SMFs group was significantly greater than that in the force‐only group at this early time point. By the fourteenth day, the PDL width in the SMFs‐treated group had approached the baseline level and remained at the baseline level by the twenty‐eighth day, while the PDL in the force‐only group was still under continuous pressure (Figure 2d,e). No abnormal resorption was observed in the apical area in either group. Additionally, more pronounced osteoclast resorption lacunae were visible on the surface of the alveolar bone in the Force+SMFs group (Figure 2d). This phenomenon occurred because the initial application of orthodontic force will compress the periodontal ligament, causing its width to decrease; subsequently, the resorption of alveolar bone mediated by osteoclasts can partially alleviate the compression of the PDL, allowing its width to recover [28]. In this study, the PDL width in the Force+SMFs group increased relatively, suggesting that SMFs effectively enhanced the osteoclastic remodeling activity, thereby reducing the continuous compression of the PDL (Figure 2d,e). Besides, the amount of TRAP+ osteoclasts within the compression side was predominantly upregulated by application of SMFs (Figure S2b,c). Taken together, these results verified that SMFs are capable of acceleration of tooth movement and bone remodeling during orthodontic treatment.

**FIGURE 2:**
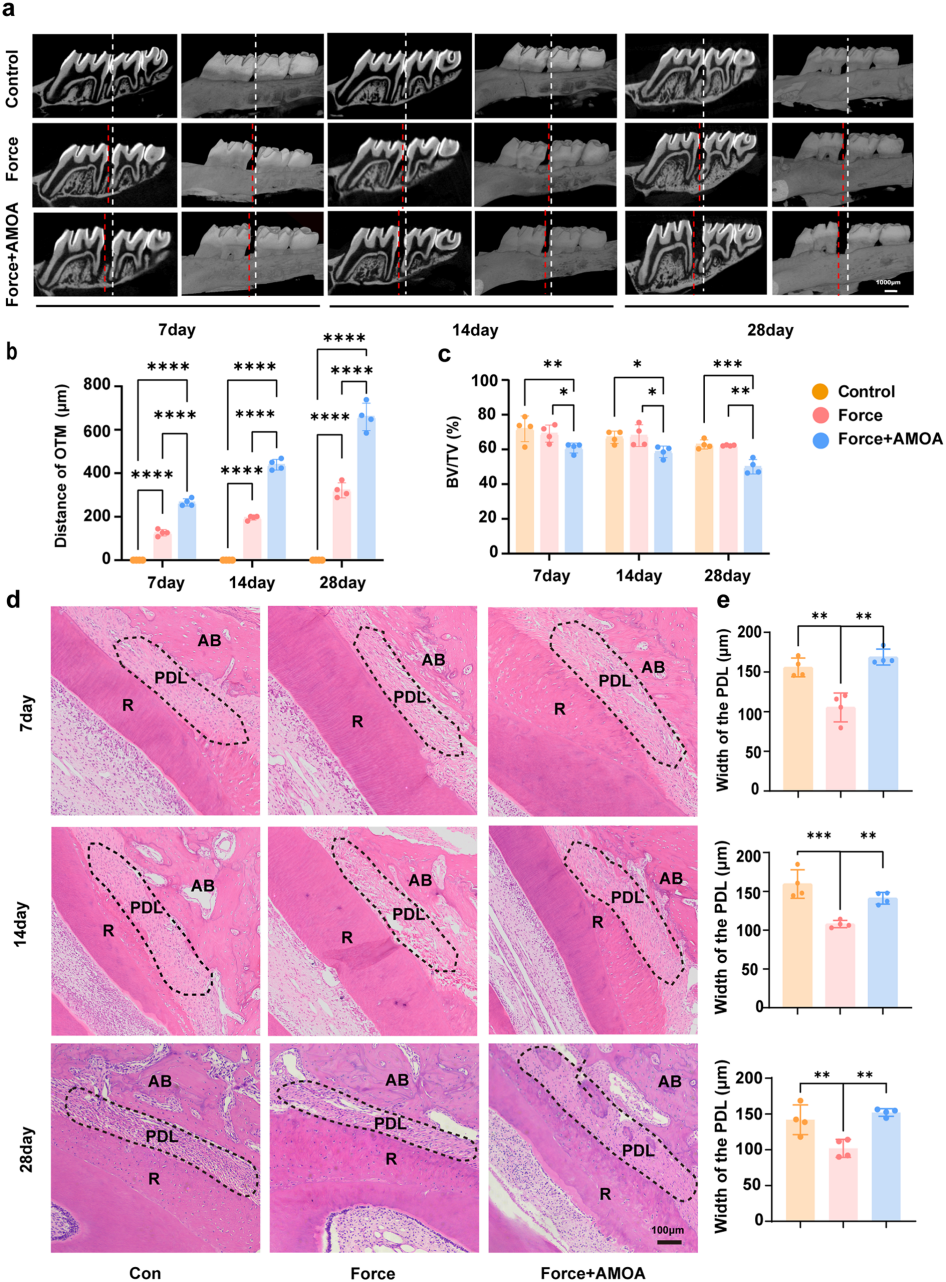
AMOA increases the movement distance of orthodontic tooth and accelerates alveolar bone remodeling. (a) Representative micro‐CT scan images of the molar region in the maxillae obtained after 7days,14 days and 28 days. Scale bar: 1000 µm. (b) Distance measurement statistic between the maxillary first molar and second molar. Mean ± SD. *n*  =  4. ^****^ *p* < 0.0001. (c) Bone volume to tissue volume ratio (BV/TV), Mean ± SD. *n*  =  4. ^*^ *p* < 0.5, ^**^ *p* < 0.01, ^***^ *p* < 0.001 (d) Hematoxylin and eosin (H&E) staining images. R: root, PDL: periodontal ligament, AB: alveolar bone. Scale bar: 100 µm. (e) Statistics of the width of the periodontal ligament. Mean ± SD. *n*  =  4. ^**^ *p* < 0.01. ^***^ *p* < 0.001.

Pro‐inflammatory activities have been widely acknowledged to dominate the OTM process [29]. Cathepsins are main participants in bone remodeling during aseptic inflammation. In the present study, Cathepsin K (CTSK) expression in the alveolar bone region was evaluated at 7, 14, and 28 days. It was already up‐regulated at day 7, increased by day 14, and remained predominantly elevated at day 28 in the AMOA group compared with controls. At day 28, this elevated CTSK was consistent with the enhanced TRAP staining observed in the same region (Figure S2b,c). In parallel, the classical pro‐inflammatory cytokines TNF‐α and IL‐1β within the PDL region displayed a similarly sustained accumulation under AMOA intervention. Their levels were evidently increased as early as day 7, peaked at day 14, and were still clearly captured at day 28 (Figure 3a, b). Furthermore, immunofluorescence analysis at day 28 revealed a marked infiltration of iNOS^+^ M1 macrophages in the PDL of the AMOA group (Figure S2f,g), suggesting an escalation of innate immune involvement in the late phase of orthodontic remodeling. Under orthodontic force, the remodeling activities within the periodontal microenvironment are primarily characterized by localized aseptic inflammation induced by immune cell activation and osteoclastic resorption. The above findings demonstrate that under SMF stimulation, both pro‐inflammatory cytokine accumulation and osteoclastic resorption activity are significantly enhanced throughout the 7‐ to 28‐day observation period, with pronounced M1 macrophage polarization observed at day 28. This indicates a persistent and intensifying immune–bone remodeling response, reflecting a more vigorous orthodontic periodontal remodeling process that ultimately accelerates tooth movement (Figure 3c).

**FIGURE 3:**
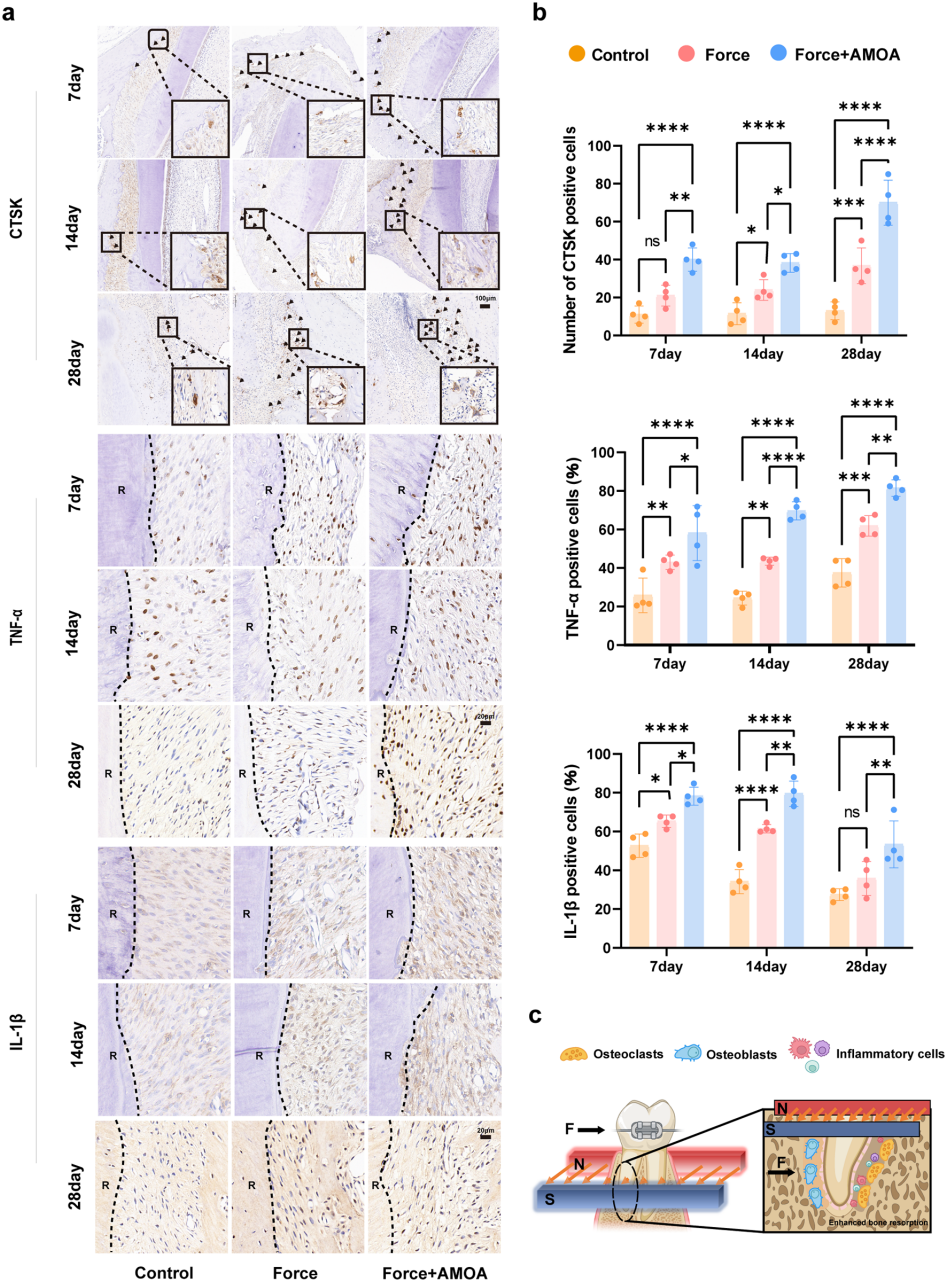
AMOA promotes osteoclast and inflammatory remodeling in periodontal area. (a) Representative images of immunohistochemical staining for CTSK, TNF‐α, IL‐1β. CTSK staining at the periodontal ligament‑alveolar bone interface. Scale bar: 100 µm(CTSK), 20 µm(TNF‐α), and 20 µm(IL‐1β). (b) Statistics of numbers of CTSK positive cells in the alveolar bone and percentage of TNF‐α, IL‐1β positive cells per field in the periodontal ligament of the first molar. Mean ± SD. *n*  =  4. ^*^ *p* < 0.05, ^**^ *p* <  0.01, ^***^ *p* < 0.001, ^****^ *p* < 0.001. (c) Diagram of the possible mechanism by which static magnetic field accelerates orthodontic tooth movement.

### 2.3 PDLCs Facilitate Pro‐Inflammatory Activation and Osteoclastic Differentiation in Paracrine Manner Owing to SMFs

To verify the above phenomenon observed in vivo, we thus constructed the cell experimental system in vitro. As PDLCs served as main participants within periodontal regions for sensing orthodontic force and physical cues [30], compressive force was vertically applied onto PDLCs to mimic orthodontic force, along with loading of static magnets for formation of SMFs. Later, isolation of culturing supernatants was performed to intervene macrophage polarization and osteoclastic differentiation (Figure 4a). To determine the indirect role of SMFs on polarization, we detected the classical M1 marker CD86 and M2 marker CD206 by fluorescent staining and flow cytometry, which all indicated an increasing tendency of M1 polarization (Figure 4b, c, d). In addition, significant increase in M1‐related cytokines, including IL‐1b, IL‐23 and IL‐12, was observed by PCR analysis, confirming that SMFs promote PDLC‐mediated macrophage inflammatory polarization (Figure 4e). To clarify the relationship between SMFs and osteoclastic differentiation, we performed Transwell assay with supernatant addition, and results showed that SMFs enhanced migration of monocytes into periodontal regions (Figure 4g). Meanwhile, expression of osteoclastic genes, such as OSCAR, DCSTAMP and TRAP, were revealed to be upregulated, altogether suggesting its effect in osteoclastic promotion (Figure 4f). In conclusion, these data supported that SMFs enhanced PDLCs‐mediated facilitation of pro‐inflammatory polarization and osteoclast differentiation in a paracrine manner.

**FIGURE 4:**
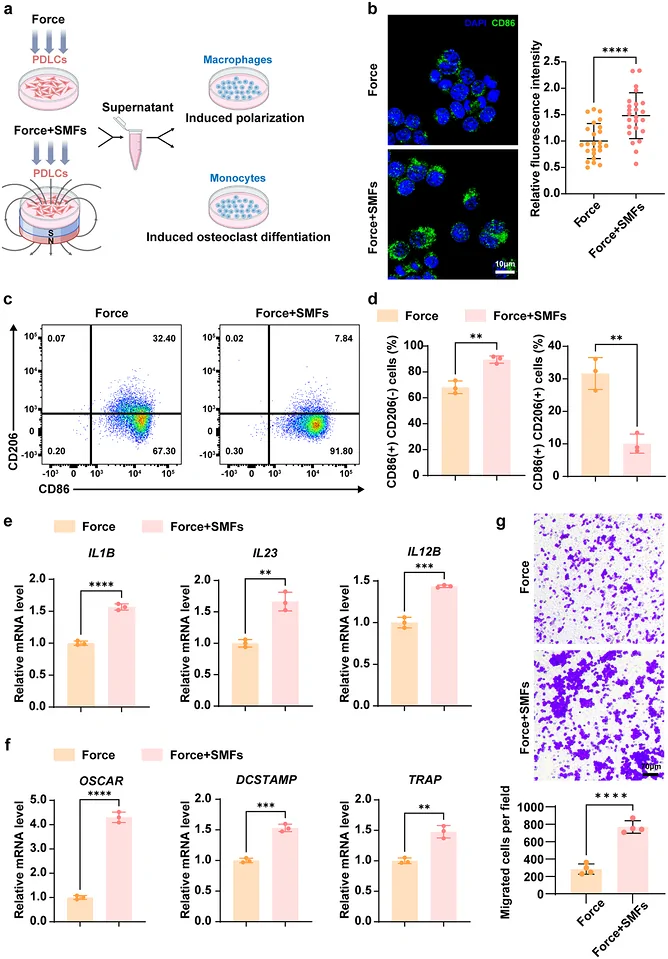
SMFs promote osteoclastic differentiation and macrophage pro‐inflammatory polarization through paracrine manner. (a) Schematic diagram of PDLCs loaded with mechanical force and SMFs in vitro and subsequent collection of supernatants for co‐culture. (b) Results of immunofluorescence staining of macrophages after 24 h of co‐culture. Scale bar: 10 µm. Mean ± SD. *n*  =  24. ^****^ *p* < 0.0001. (c)(d) Flow cytometry results of macrophages after 24 h of co‐culture. Mean ± SD. *n*  =  3. ^**^ *p* < 0.01. (e) qRT‐PCR results of *IL1B, IL23* and *IL12B* expression in macrophages after 24 h of co‐culture. Mean ± SD. *n*  =  3. ^**^ *p* < 0.01, ^***^ *p* < 0.001, ^****^ *p* < 0.0001. (f) qRT‐PCR results of *OSCAR, DCSTAMP* and *TRAP* expression in monocytes after 24 h of co‐culture. Mean ± SD. *n*  =  3. ^**^ *p* < 0.01, ^***^ *p* < 0.001, ^****^ *p* < 0.0001. (g) Results of crystal violet staining of monocytes after 24 h of co‐culture in the migration assay. Scale bar: 70 µm. Mean ± SD. *n*  =  4. ^****^ *p* < 0.0001.

### 2.4 SMFs Promote Spreading Behavior and Cell‐Matrix Interaction of PDLCs for Orthodontic Mechanical Signaling

To explore the mechanistic explanation of SMFs‐induced functional regulation on PDLCs, we cultured PDLCs for initial 6 h to obtain early cell adhesion, then subjected them to compressive force with or without SMFs for 12 or 24 h. A static control group (no force, no SMFs) was included at each time point. Immunostaining for Vinculin at both 12 and 24 h revealed that the area of vinculin‐containing focal adhesions (FAs) per cell was significantly larger in the force‐only group compared with the static control, and was further enlarged in the AMOA group (Figure 5a,c). Consistently, F‐actin staining showed that cell spreading area followed the same pattern: force alone increased spreading relative to the control, and AMOA induced an additional increase at both time points (Figure 5a,b). In contrast, the cell shape index did not differ between the static control and the force‐only group, but was significantly lower in the AMOA group than in both of the other groups, indicating a more elongated morphology specifically driven by SMFs (Figure 5d). After confirming the enhanced cell spreading under SMFs, we sought the underlying mechanisms. Tension generated and transmitted through the cytoskeleton is exerted across FAs to form integrin‐mediated matrix interactions. Immunofluorescence analysis demonstrated that, at both 12 and 24 h, FAK expression and its enrichment at FAs were comparable between the static control and the force‐only group. However, exposure to SMFs (AMOA group) led to a significant upregulation of FAK and its pronounced spatial accumulation within FAs (Figure 5e,f), suggesting that SMFs specifically activate FAK signaling to reinforce focal adhesions. Downstream of FA adhesion, intracellular mechano‐transduction via YAP/TAZ was examined. YAP/TAZ nuclear translocation was observed in all groups, but the nuclear abundance increased stepwise: the force‐only group showed significantly higher nuclear YAP/TAZ than the static control, and the AMOA group displayed a further significant elevation compared with the force‐only group at both 12 and 24 h (Figure 5g). This was quantitatively confirmed by the nuclear‐cytoplasmic ratio of YAP/TAZ (Figure 5h). Together, these data demonstrate that SMFs enhance cell spreading and mechano‐transduction by specifically upregulating FAK signaling and promoting focal adhesion growth, and induce an elongated morphology. These effects are already evident as early as 12 h and persist through 24 h, indicating a rapid and sustained mechanobiological response of PDLCs to SMFs under compressive loading.

**FIGURE 5:**
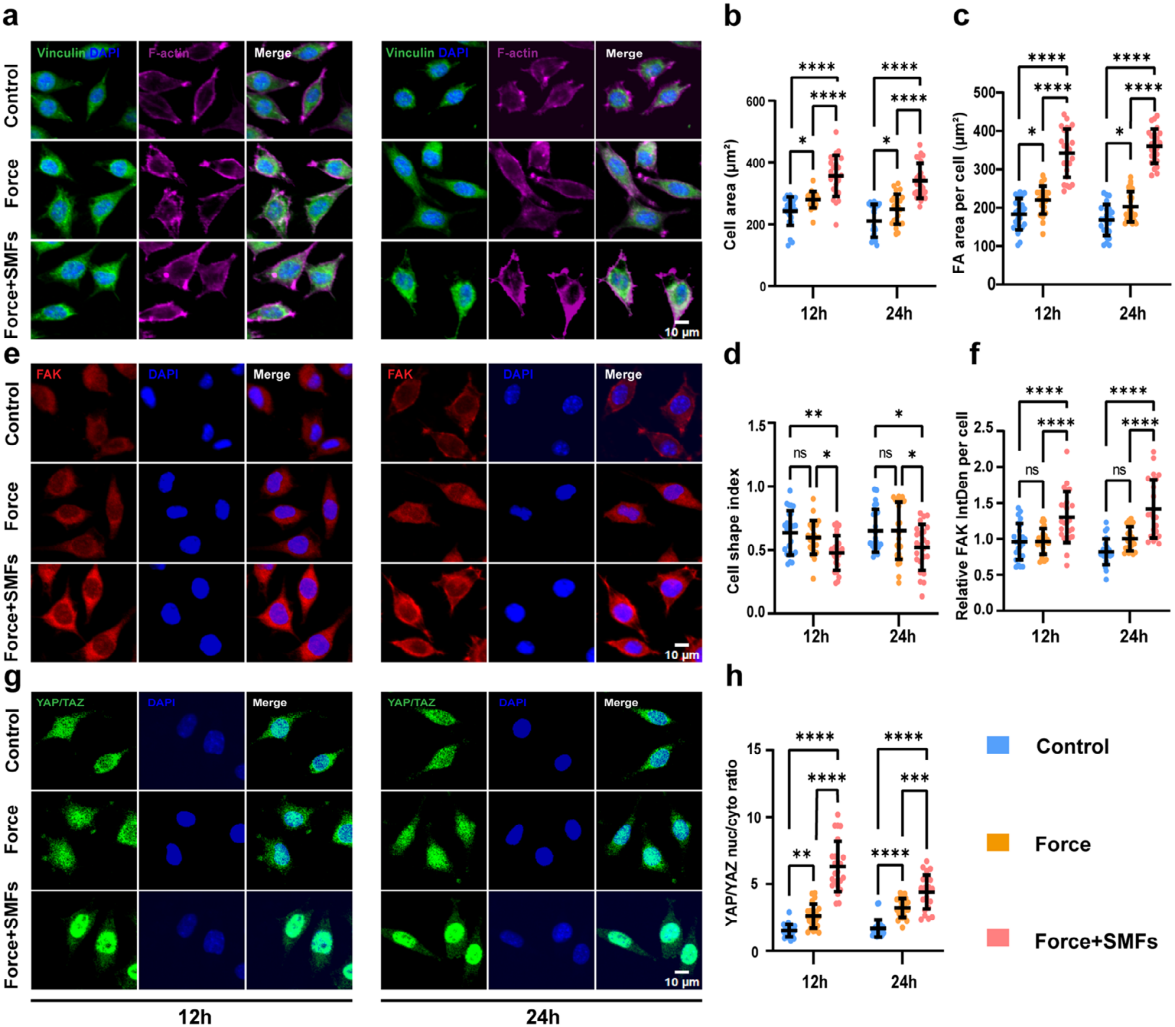
SMFs amplify the response of orthodontic mechanical signaling pathways. (a) Representative immunofluorescence images of vinculin (green), F‐actin (purple), and nuclei (blue) in PDLCs under three conditions: control (without mechanical force and without SMFs), with mechanical force only, and with mechanical force plus SMFs. Scale bar: 10 µm. (b) Cell area, (c) FA area per cell, and (d) cell shape index in PDLCs. Mean ± SD. *n*  =  24. ^*^ *p* < 0.05, ^**^ *p* < 0.01, ^****^ *p* < 0.0001. (e) FAK: Focal Adhesion Kinase. It is a mechanotransducer that becomes activated upon orthodontic force, regulating downstream bone remodeling and inflammatory responses in PDLCs. Representative immunofluorescence images of FAK (red) and nuclei (blue) in PDLCs under three conditions: control (without mechanical force and without SMFs), mechanical force only, and mechanical force plus SMFs. Scale bar: 10 µm. (f) Relative FAK integrated density per cell in PDLCs. Mean ± SD. *n*  =  24. ^****^ *p* < 0.0001. (g) Representative immunofluorescence images of YAP/TAZ (green) and nuclei (blue) in PDLCs under three conditions: control (without mechanical force and without SMFs), mechanical force only, and mechanical force plus SMFs. Scale bar: 10 µm. (h) The nuclear‐cytoplasmic ratios of YAP/TAZ in PDLCs analyzed from the immunofluorescence images. Mean ± SD. *n*  =  24. ^**^ *p* < 0.01, ^***^ *p* < 0.001, ^****^ *p* < 0.0001.

### 2.5 SMFs Enlarge Orthodontic Mechano‐Transduction Through Activation in Microtubule‐Integrin Assembly

To elucidate the mechanism for SMFs‐mediated activation in mechano‐transduction in advance, we performed bulk RNA sequencing for high‐throughput analysis (Figure 6a). Interestingly, we found that differentially expressed genes were mostly involved in immune response, microtubule movement and stress response (Figure 6b). Similarly, KEGG enrichment analysis also revealed pathways associated with immune activity (IL‐17 signaling, TNF signaling, etc.) and microtubule function (motor protein and MAPK signaling) (Figure 6c). Therefore, we first focused on enhanced secretion of pro‐inflammatory cytokines by SMFs influence, which were validated by upregulated expression levels of CCL7, TNF and IL‐6 in RNA sequencing, and by qRT‐PCR showing that IFNγ, M‐CSF and TNF were significantly elevated at both 12 and 24 h under SMFs treatment compared with the force‐only group (Figure 6d,e). These data indicated that SMFs‐mediated paracrine manner observed above might be explained by facilitation in production of a panel of pro‐inflammatory cytokines. Subsequently, attention was attracted by changes in microtubule function under SMFs intervention, as integrin‐mediated focal adhesions has been reported to be largely dependent on accumulation and assembly with microtubule [31, 32]. Crucial proteins of integrin family were elevated by SMFs, explaining the strengthened orthodontic mechano‐transduction observed above. Importantly, Fluorescent staining confirmed predominant accumulation of a‐Tubulin proteins in focal adhesions (Figure 6f), along with co‐localization of a‐Tubulin and Integrin‐β1 evidenced by static analysis (Figure 6g). As fundamental crosstalk between microtubules and integrins could contribute to cellular mechano‐transduction [33], these data supported that SMFs facilitated orthodontic mechano‐sensitivity through activation of microtubule assembly.

**FIGURE 6:**
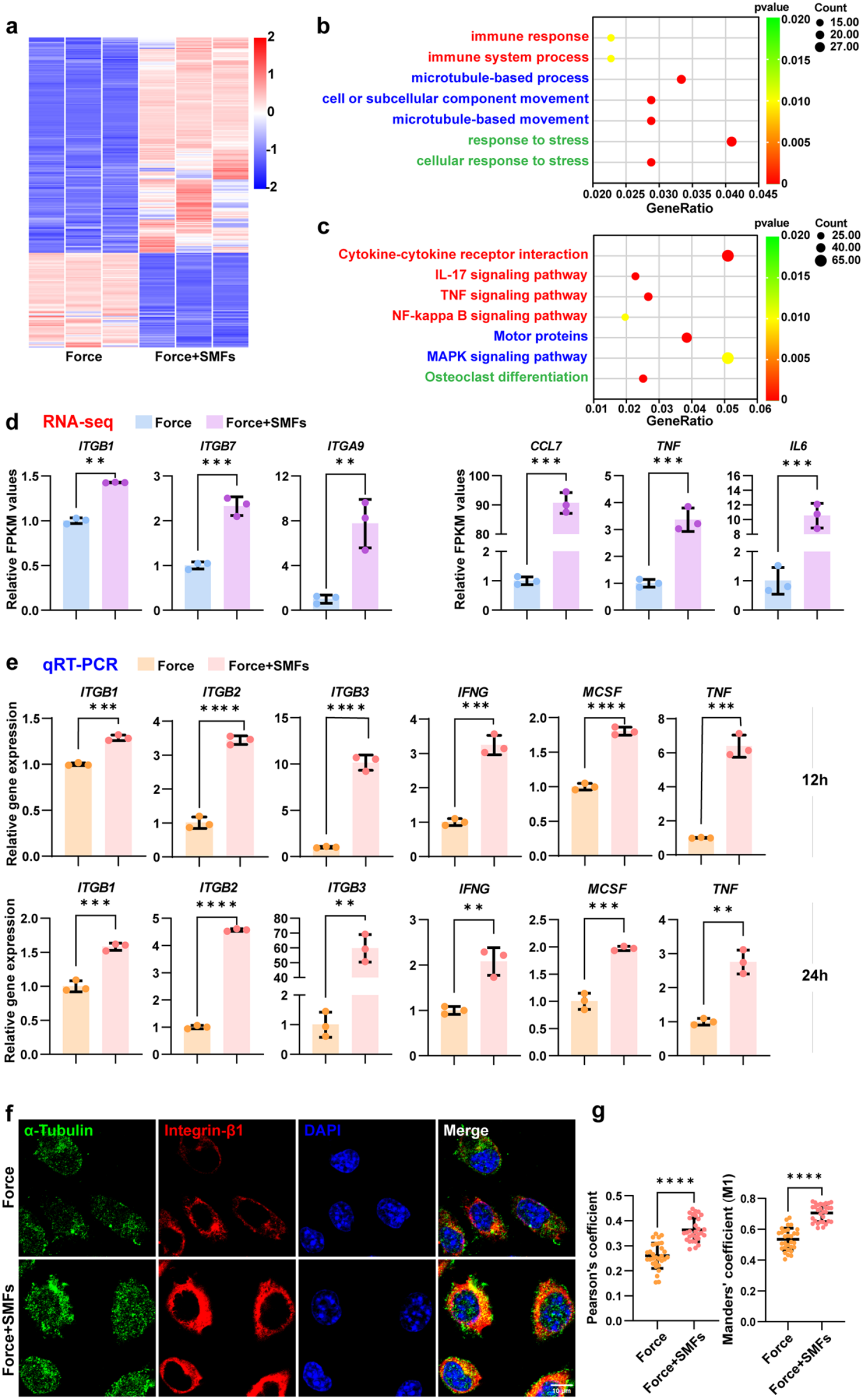
SMFs enhance orthodontic mechano‐transduction via activation in microtubule‐integrin assembly of PDLCs. (a) Heat map for cluster analysis of differentially expressed genes (DEGs). (b) Bubble plot of GO enrichment results of DEGs. (c) Bubble plot of KEGG enrichment results of DEGs. (d) Relative FPKM values of *ITGB1, ITGB7, ITGB9, CCL7, TNF* and *IL6* from RNA‐seq data. Mean ± SD. *n*  =  3.  ^**^ *p* < 0.01,  ^***^ *p* < 0.001. (e) 12h and 24h Relative gene expression of *ITGB1, ITGB2, ITGB3, IFNG, MCSF* and *TNF* from qRT‐PCR. Mean ± SD. *n*  =  3.  ^**^ *p* < 0.01,  ^***^ *p* < 0.001,  ^****^ *p* < 0.0001. (f) Representative immunofluorescence images of α‐Tubulin (green), Integrin‐β1 (red) (merged yellow) and nuclei (blue) in mechanical force applied PDLCs without and with SMFs. Scale bar: 10 µm. (g) Pearson's coefficient and Manders’ overlap coefficient (M1) for the colocalization analysis between α‐Tubulin and Integrin‐β1. Mean ± SD. *n*  =  30.  ^****^ *p* < 0.0001.

### 2.6 Fabrication and Pre‐Clinical Evaluation of AMOA Device

Based on the above systemic study about experimental AMOA, efforts were further made to explore the potential of AMOA in clinical application. Patient anatomic morphology was captured via oral scanning, which forms the basis for AMOA design and fabrication (Figure 7a). On dependence of the human anatomic features, the AMOA consists of three segments of magnetic filling on the buccal and lingual flange respectively to controllably cover the whole dentition (Figure 7b,c). Combination of dynamic simulating and 3D magnetic filling techniques ensures accurate formation of local magnetic field (Figure 7d–f), which is necessary for customized local utilization of magnetic field. Using standard emulating dental model, we confirmed that dressing of AMOA device into oral cavity is practicable (Figure 7g). Based on this, 10 patients receiving standard orthodontic treatment were recruited in our clinical center. Upon accepting the experimental agreement, AMOA device is developed following the above procedures in Figure 7h. Volunteers were required to get AMOA dressed in their dentition for 30 min every day. After 20‐day period, clinical information about device utilization were collected for analysis. Results showed that 80% volunteers exhibited subjective comfort for using AMOAs (Figure 7i). 2 patients reflected a slight feeling of crowding, which might be solved by improvement on 3D modeling process. A small increase in salivary flow was observed in some patients within ten minutes of initial use, but this phenomenon gradually became less evident with subsequent use (Figure 7j). Clinical examination further ensured that no abnormal or allergic reactions appeared in oral mucosa tissues after AMOA dressing, as well as no visible change in teeth (Figure 7k). These results altogether served as basis of biocompatibility and feasibility before test on its clinical effect. We are planning to further detect its role in orthodontic acceleration clinically in the future.

**FIGURE 7:**
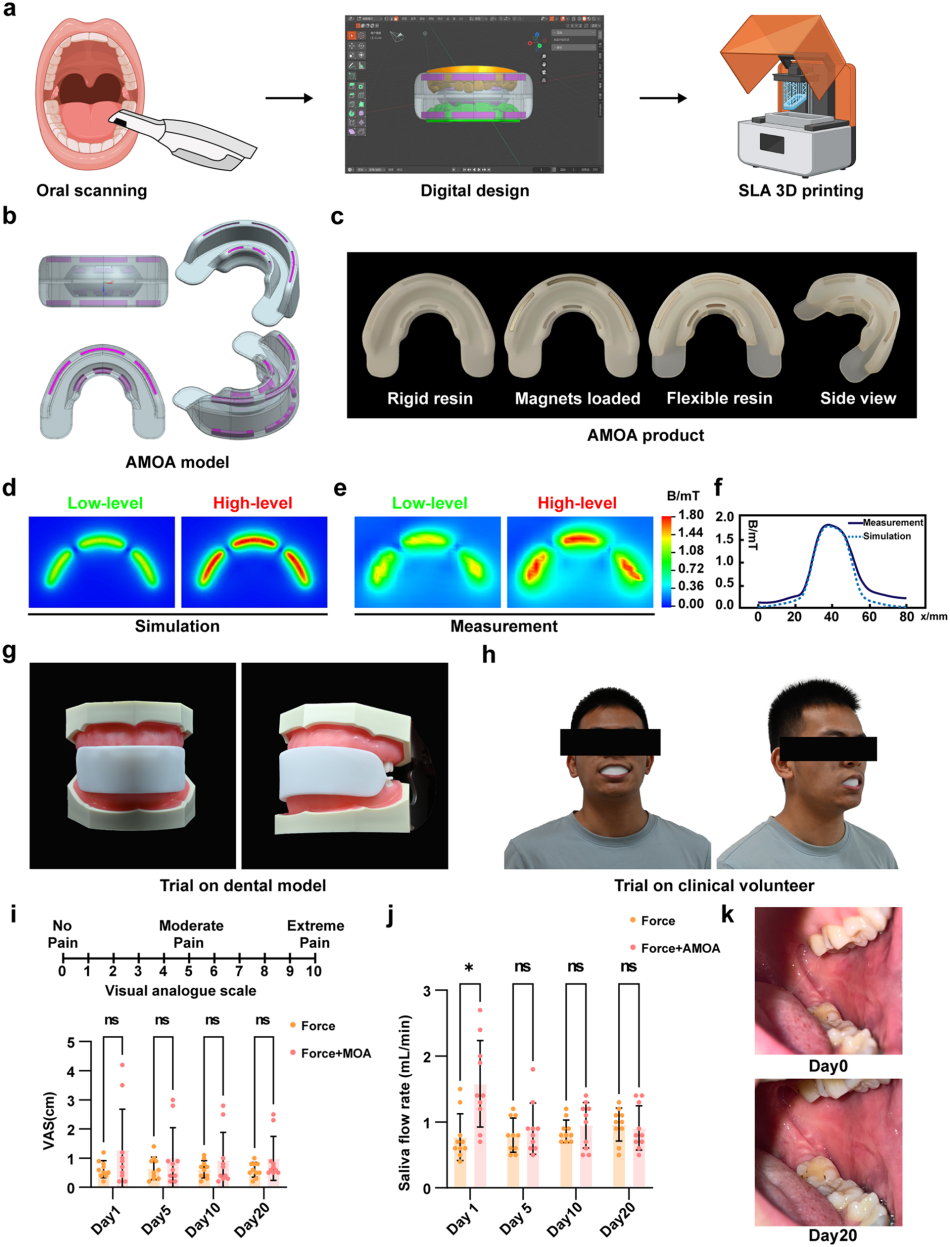
Pre‐clinical evaluation of AMOA device. (a) Oral scanning and dental arch reconstruction were used for the AMOA model design and SLA printing. (b) The model design of AMOA. (c) The finished product of AMOA in rigid and flexible resin, then loaded with arrayed magnets. (d) Expected magnetic field strength under simulation. (e) The magnetic field strength obtained from the actual measurement. (f) Image of the fitting function for the relationship between magnetic field strength and horizontal distance. (g) Images of the oral model wearing the AMOA. (h) Images of one clinical study volunteer wearing the AMOA. (i) Comfort assessment results by using a visual analogue scale and (j) saliva flow rate of orthodontic patients with or without the application of AMOA on day 1, day 5, day 10 and day20. Mean ± SD. n  =  10. ns, not significant, ^*^ *p* < 0.05. (k) Images of the buccal mucosa of one clinical study volunteer using AMOA on day 0 and day 20. No significant ulcers or redness was observed in any of the volunteers.

## 3 Conclusions

This study proposed a customized and adjustable orthodontic auxiliary accelerator by precise local exertion of static magnetic field within periodontal regions. Combination of phase‐field simulation and addictive manufacturing ensures accurate molding of AMOAs and precise localization of static magnetic effect. The synergistic role of arrayed magnetic field application and orthodontic force significantly enhanced tooth movement efficiency by 105%. Mechanistically, static magnetic effect could amplify the sensing of biomechanical signals of PDLCs, thus strengthening intracellular signals. As a result, remodel of periodontal microenvironment was driven via paracrine manners of PDLCs, evidenced by enhanced inflammatory macrophage polarization and upregulated number of osteoclasts. These altogether promoted remodeling of alveolar bone tissue and accelerated orthodontic tooth movement. In summary, this exploration on AMOA‐mediated high‐performance orthodontic treatment is anticipated to offer basic reference and rationale for clinical transformation of AMOA to achieve light‐force, healthy and efficient orthodontics.

## 4 Discussion

Fast and short‐term tooth movement using low‐level force loading is the ideal situation for orthodontic treatment, as it reduces redundant intervention course and avoid potential adverse complication risks. Nevertheless, this target fails to come true. It is mainly explained by the fact that simple force intervention is unable to actuate fast bone metabolism, resulted from the limitation of mechanical sensing in resident PDLCs. Biophysical cues, including heat, electricity, light and magnetic field have been validated to regulate biological functions during tissue reconstruction [34, 35, 36]. However, which and how these biophysical cues could facilitate orthodontic bone remodeling still remains unclear. Therefore, development of novel orthodontic auxiliary technology and elucidation of its inherent mechanisms indicate significant clinical value.

COMSOL multi‐physical simulation, a classical tool for analyzing physical phenomenon and presenting visible screen was first performed to simulate spatial distribution of arrayed magnetic field. This result could precisely guide our design and molding of AMOA to generate indicated SMFs within targeted periodontal regions. Then, based on high‐accuracy 3D printing platform, the personalized AMOA with controllable magnetic field was successfully fabricated, which allows individual matching of oral anatomic landscape and precise exertion of magnetic field for efficient biological intervention.

Our study validates the local static magnetic field at 30 mT could produce high‐performance synergistic effect in force‐induced orthodontic tooth movements for the first time. Compared with other physical cues including light, heat and ultrasound, magnetic field shows scarce energy dissipation when penetrating tissues, making it an ideal choice for deep region intervention, especially bone. The role and parameter of magnetic effect in bone repair and bone fracture healing have been explored [37], but how magnetic field could affect orthodontic process has not been elucidated yet. Considering the increased vessel density and metabolic rate of alveolar bone compared with long bones, the optimum magnetic field at 30 mT for orthodontics significantly differs from that at 100–200mT for bone repair. In addition, orthodontic treatment is accompanied by biological force loading, while bone healing relies on a static period, which could partially explain the difference in their optimum parameters.

Mechanistic exploration delineates that magnetic effect drives remodeling of microenvironment via strengthening the transducing and sensing of mechanical signals from orthodontic loading. It has been elucidated that biological force during orthodontic treatment is mainly perceived by PDLCs located within periodontal ligaments [12]. Mechanical signals are subsequently transformed into intracellular messages for activation of PDLCs, thus leading to a series of downstream biological reactions including pro‐inflammatory and osteoclastic responses for tooth movements. However, the efficiency of orthodontic treatment is restricted by the sensitivity and adaptability of PDLCs for mechanical sensing. That is to say, weak force fails to trigger periodontal remodeling, while increase in force loading could not result in equivalent tooth movements. In addition, overloading of mechanical force would induce severe side effects including adsorption of tooth roots and excessive destruction of alveolar bone [4, 5]. Our findings identify SMFs as a “mechanical sensitizer” that enhances PDLC mechanosensitivity and amplifies downstream biological responses. This metabolic activation accelerates PDLC‐mediated periodontal remodeling, evidenced by significantly promoted orthodontic movement in a 4‐week rodent model.

The primary biophysical trigger involves a radical pair mechanism (RPM) operating on tubulin subunits during GTP hydrolysis, which modulate microtubule polymerization rates [38], together with the diamagnetic anisotropy of tubulin that aligns polymerizing microtubules along the SMF direction. These microtubule alterations promote integrin clustering and focal adhesion conformation, thereby initiating the downstream FAK‑YAP signaling cascade.Compared with invasive surgical techniques (corticotomy, piezocision, micro‑osteoperforations) and non‑invasive modalities (low‑level laser therapy, vibration), the AMOA is non‑invasive, patient‑applied, requires only 30 min/day, and is portable. Unlike surgical methods, it causes no tissue trauma; unlike LLLT, it needs no specialized equipment or repeated office visits.

When translating to humans, species differences matter: rodent bone remodeling occurs in weeks, human in months. Thus, the acceleration magnitude observed here may overestimate absolute clinical efficacy. However, the conserved microtube‐integrin‐FAK‐YAP pathways provide a strong biological rationale, though the relative acceleration is expected to be more modest.Device positioning requires precision (achievable via 3D printing); patient compliance is favored by the short daily wear, but the removable design risks inconsistent use. The 30 mT SMF is within the safe range, with minimal cumulative risk. Future studies need large‐animal validation (e.g., canines) and human trials to quantify acceleration and long‐term safety (gingival health, root resorption, pulp vitality).

## 5 Materials and Methods

### 5.1 Animals

Male Sprague‐Dawley (SD) rats (weight, 180 to 200 g; age, 6 weeks) were obtained from Beijing HFK Bioscience Co. Ltd. (Beijing, China). Rats were housed in cages (3 rats/cage) under automatically controlled conditions of temperature (23°C ± 1°C), humidity (50% ± 10%), and a 12:12‐h light‐dark cycle. They were given tap water and standard laboratory chow ad libitum. This study was approved by the Ethics Committee for Animal Experiments at the Tongji Medical College ([2024] IACUC Number: 4153) and conformed with the ARRIVE 2.0 guidelines for preclinical studies.

### 5.2 Design and Fabrication of AMOA

The internal 3D structures of the oral cavity in animal models and human participants were acquired using micro‐computed tomography (Micro‐CT) and intraoral scanning, respectively. The scanned data were exported in Standard Tessellation Language (STL) format and imported into 3D reverse engineering software (Geomagic Freeform, 3D Systems, USA) for morphological optimization—including mesh repair, smoothing, and anatomical enhancement—to reconstruct a precise digital model of the oral environment. Based on this model, a personalized magnetic accelerator was designed in computer‐aided design (CAD) software (SolidWorks 2022, Dassault Systèmes, France). The design incorporated predefined slots to house N52‐grade neodymium iron boron (NdFeB) magnets with a thickness of 1 mm. To achieve a concentrated static magnetic field across the PDL, the magnets were arranged in a “sandwich” configuration: one magnet positioned on the buccal side and another on the palatal side of the target tooth (rat maxillary first molar). This arrangement ensures that the magnetic field directly traverses the mechanosensitive PDL and alveolar bone, while the mutual attraction between the two magnets stabilizes the appliance. The spatial arrangement and polarity orientation of the magnets were informed by finite element simulations of magnetic field distribution performed in COMSOL Multiphysics (v6.1, COMSOL AB, Sweden) using the AC/DC Module and static magnetic field solver. The simulations predicted a static magnetic field intensity of approximately 30 mT at the tooth surface. We selected a static (rather than pulsed) magnetic field because static fields are more portable, free from thermal/electrical effects, and suitable for long‐term continuous exposure in orthodontic applications. The chosen intensity (30 mT) falls within the moderate range (1 mT–1 T) and is consistent with studies showing that 20–100 mT static magnetic fields effectively enhance bone remodeling without cytotoxicity [39, 40]. The accelerator devices were then fabricated via stereolithography (SLA) on a high‐precision 3D printer (Formlabs Form 3+, USA) using a biocompatible acrylate resin. After printing, the devices were washed in isopropanol, UV post‐cured, and polished according to manufacturer protocols. The NdFeB magnets were securely affixed into the slots using a medical‐grade epoxy adhesive. Finally, the actual magnetic field distribution of the assembled device was experimentally measured using a gaussmeter (model: HM‐3000, Hangsung Inc., Korea) to validate the simulation results and ensure functional performance.

### 5.3 Orthodontic Force and AMOA Application

A total of 36 SD rats were randomly divided into three group, with 12 rats in each group. Animals were anesthetized with isoflurane gas (2%) using small animal anesthesia machine (RWD‐R500). 0.2‐mm nickel‐titanium coil springs (Xihubiom), each with an activating force of approximately 50 grams (Figure S1a) [41], were installed to connect the maxillary first molars to the maxillary incisors. This force level enables rats to achieve maximum tooth movement with acceptable root resorption. We used a dynamometer (HANDPI) to conduct daily measurements of the force magnitude throughout the 28‐day experimental period. SLA 3D‐printed AMOAs were used to deliver a static magnetic field of ∼30mT on the buccal and palatal periodontal regions of the first maxillary molars in Force + AMOA group. For the blank control group, the same teeth were connected with springs, but with no force applied. Maxillary specimens were harvested from four randomly selected rats per group (n = 4) on experimental days 7, 14, and 28.

### 5.4 Micro–Computed Tomography (CT) Scanning

The maxillaries of rats were scanned using a Skyscan 1076 micro‐CT (Inveon Multimodality Scanner; Siemens, Erlangen, Germany) at 70 kV, 350 µA, 180° rotation, 0.3° rotation step, and a resolution of 9 µm. Subsequently, three‐dimensional images of the specimens were reconstructed from the scan data utilizing the micro‐CT system's software package. The tissue between the first molar roots in each group was measured to calculate the OTM‐related parameters, bone volume to total volume (BV/TV), trabecular bone separation (Tb. Sp) and trabecular bone thickness (Tb. Th) to evaluate the alveolar bone remodeling. The orthodontic tooth movement (OTM) distance was defined as the linear distance between the closest contact points of the first molar (M1) and the corresponding homolateral second molar (M2), in accordance with methodologies described in other pertinent studies [42, 43, 44].

### 5.5 Histology and Tartrate‐Resistant Acid Phosphatase (TRAP) Staining

The maxillary specimens were collected, fixed in 4% paraformaldehyde, demineralized in 15% ethylenediaminetetraacetic acid (EDTA), and then embedded by paraffin. Longitudinal serial sections (4µm) were obtained from the maxillary molar regions of each group. Hematoxylin–eosin (HE) staining was used for descriptive histology, whereas TRAP staining was conducted to detect osteoclasts using a leukocyte acid phosphatase kit (387A, Sigma).

### 5.6 Immunohistochemistry

Immunohistochemistry was performed with the Immunohistochemistry (IHC) Detect Kit for Rabbit/Mouse Primary Antibody (Proteintech, PK10006). Primary antibodies against rat CTSK (Proteintech, 11239‐1‐AP, 1:200), TNF‐α (Proteintech, 60291‐1‐Ig, 1:200) and IL‐1β (Proteintech, 26048‐1‐AP, 1:200) were used.

### 5.7 Immunofluorescence on Histological Sections

The processing and sectioning of the maxillary specimens were carried out in accordance with the method previously described. The tissue sections were deparaffinized and rehydrated, followed by immersion in an antigen retrieval buffer (pH 6.0) containing citrate. Antigen retrieval was performed using a microwave oven. Upon cooling, the slides were subjected to three consecutive washes in phosphate‐buffered saline (PBS) for 5 min each. Subsequently, the sections were blocked with 5% bovine serum albumin (BSA) and incubated overnight at 4°C with the following primary antibodies, respectively, at 4°C overnight: anti‐CD68 (Bio‐Rad, MCA341R, 1:500), anti‐iNOS (Abclonal, A3774, 1:200). After washing with PBS three times, the sections were incubated with CoraLite488‐conjugated secondary antibody or cy3‐conjugated secondary antibody specific to the species of the primary antibodies (Proteintech, 1:200), for 1 h at room temperature in the dark. DAPI was used for nuclear counterstaining. The sections were then visualized using a confocal microscope (Nikon A1‐Si, Japan).

### 5.8 Human Primary Periodontal Ligament Cells (PDLCs) Culture

The protocol to obtain primary periodontal ligament cells (PDLCs) was approved by the Institutional Research Ethics Committee of Tongji Medical College (Wuhan, China). Informed consent was obtained from the orthodontic patients prior to extraction. A total of four cases of extracted premolars (based on the orthodontic treatment plan) were collected from patients under the age of 25 with dental‐maxillofacial deformities, at the Union Hospital of Tongji Medical College, Huazhong University of Science and Technology (Wuhan, China). PDL was gently separated from the surface of the root and then digested in a solution of 3 mg/mL collagenase type I (Biosharp) and 4 mg/mL dispase (Coolaber) for 1 h at 37°C. PDLCs were cultured in the alpha modification of Eagle's medium (α‐MEM) containing 15% v/v fetal calf serum (Thermo Fisher Scientific), 100 U mL−1 penicillin and 100 g mL−1 streptomycin at 37°C in a humidified atmosphere with 5% CO2 and then were used at passage 4.

### 5.9 In Vitro Compression Force, SMFs Application and Indirect Coculture System

PDLCs were plated at a density of 1*105 cells per well in 6‐well plates. When the cultured cells reached 70% to 80% confluence, we used a bionic pressure cell culture apparatus (Naturethink) to achieve a compressive force of 2.5 mmHg for 24h vertically applied onto cells (Figure S3b). For the Force + SMFs group, we used neodymium magnets to deliver a static magnetic field of ∼30mT for another 24h (Figure S3a). After that, the supernatants were collected and used for indirect coculture with macrophages and monocytes.

For indirect coculturing, THP‐1 cells were initially seeded into the plates with PMA (100 ng L^−1^; Selleck Chemicals) for 24 h. Subsequently, the culture medium was replaced with supernatants derived from PDLCs. Similarly, when seeded onto the plates for 24 h, the culture medium of THP‐1 cells was subsequently substituted with PDLC‐derived supernatants.

### 5.10 Transwell Cell Migration Assay

200 µL of a cell solution containing 4×10^4 cells were carefully pipetted onto the membrane of a 24‐well Transwell insert (LABSELECCT, 8 µm pore size), after which the insert was incubated for 10 min at 37°C and 5% CO2. Subsequently, 500 µL of PDLC‐derived supernatants were added to the well directly beneath the Transwell insert, and the plate was incubated at 37°C and 5% CO2 for 12 h. Any non‐migrated cells were gently removed from the apical side of the Transwell insert membrane using a cotton‐tipped applicator. The Transwell insert was then washed three times with PBS and fixed with 4% paraformaldehyde (PFA) for 15 min. Following this, the Transwell insert was placed into a well containing 0.2% (w/v) crystal violet and incubated for 5 min, after which it was washed with PBS or water to remove excess crystal violet. Images were obtained by a NIB610 microscope (Nexcope) and analyzed by ImageJ (NIH).

### 5.11 Cell Immunofluorescence Staining

After cocultured for 48h, macrophages were washed with PBS three times and fixed with 4% paraformaldehyde (PFA) for 20 min. Subsequently, they were blocked with 5% bovine serum albumin (BSA) for 1 h, followed by incubation with the solution of the primary antibody for CD86 (Proteintech, 13395‐1‐AP, 1:200) at 4°C overnight. The cells were then incubated with CoraLite488‐conjugated secondary antibody (Proteintech, 1:200) and nuclei were counterstained with DAPI.

PDLCs were respectively incubated with the primary antibody for Vinculin (Proteintech, 26520‐1‐Ap, 1:300), FAK (Proteintech, 12636‐1‐AP, 1:400), YAP/TAZ (Proteintech, 13584‐1‐Ap, 1:200), α‐Tubulin (Abclonal, AC012, 1:200) and Integrin‐β1 (Abclonal, A23497, 1:200). After washing with PBS three times, PDLCs were incubated with CoraLite488‐conjugated secondary antibody or cy3‐conjugated secondary antibody (Proteintech, 1:200) specific to the species of the primary antibodies.The filamentous actins were stained with phalloidin (Beyotime Biotech., Wuhan, China) and nuclei were counterstained with DAPI.

Images were obtained by a confocal laser scanning microscope (Nikon A1‐Si) with identical acquisition settings across all groups, and quantitative analysis was performed using ImageJ (NIH). For background subtraction, the rolling ball method (radius = 50 pixels) was applied to all fluorescence channels. Regions of interest (ROIs) were defined manually or via threshold‑based segmentation; for nuclear/cytoplasmic separation, the DAPI channel was used to create nuclear masks via auto‑threshold (Otsu method) followed by watershed separation to split touching nuclei. Mean fluorescence intensity was measured within each ROI by dividing the integrated density by the area, expressed in arbitrary units, after background subtraction. Area percentage was calculated for positively stained regions using a fixed intensity threshold applied uniformly across all images, computed as (positive pixel area / total tissue area) × 100%. Cell count was obtained by counting DAPI‑positive nuclei using the “Analyze Particles” function after nuclear segmentation (minimum size = 20 µm^2^, circularity = 0.5–1.0); for specific positively stained cells, the same segmentation parameters were applied to the corresponding channel, and cells were counted manually or automatically based on colocalization with nuclear staining. All quantifications were performed blinded to group allocation by two independent observers, with three randomly selected fields per section and three sections per sample, and results were averaged to obtain a representative value per group.

### 5.12 Flow Cytometry

Macrophages were quantified by flow cytometry using the APC Anti‐Mouse CD86 Antibody (Elabscience, E‐AB‐F0994E) and PE Anti‐Mouse CD206/MMR Antibody (Elabscience, E‐AB‐F1135D). In brief, 1*106 cells were collected and washed with PBS, then fixed with 4% paraformaldehyde for 20 min, followed by incubation with 1% BSA for 1 h at room temperature. Then, 5 µL of flow cytometry antibody in 100 µL of PBS was added to each sample for 30 min incubation in the dark. Subsequently, the samples were washed with PBS and analyzed by a BD FACSymphony A1 Cell Analyzer.

### 5.13 Quantitative Real‐Time Polymerase Chain Reaction (PCR)

Total RNA was isolated from cells with TRIZOL (Vazyme, Nanjing, China) according to the manufacturer's instructions and was reverse‐transcribed with the HiScript II Q RT SuperMix for qPCR kit (Vazyme, Nanjing, China) for synthesis of complementary DNA (cDNA). Real‐time polymerase chain reaction (RT‐PCR) assays were performed using SYBR Green PCR Master Mix (Vazyme, Nanjing, China) in PCR Thermocycle Instrument (Applied Biosystems). The relative expression level was analyzed by the 2−ΔΔCt method, normalized to expression of actin beta (ACTB). The primer sequences are listed as follows:

**Table:** 

Gene Name	Primer Type	Primer Sequence (5′ → 3′)
**ACTB**	**Sense**	**CACCATTGGCAATGAGCGGTTC**
	**Antisense**	**AGGTCTTTGCGGATGTCCACGT**
**IL1B**	**Sense**	**CCACAGACCTTCCAGGAGAATG**
	**Antisense**	**GTGCAGTTCAGTGATCGTACAGG**
**IL12B**	**Sense**	**GAGCCTTCTCTGCTCCCTGATA**
	**Antisense**	**GACTGAGGCTTGGAATCTGCTG**
**OSCAR**	**Sense**	**GCAGCGAGGTGCTGGTCATCA**
	**Antisense**	**ACTGCGCCAGTCAAAAGTGACC**
**DCSTAMP**	**Sense**	**CTTGCCAGGGTTTGAGGTTCAC**
	**Antisense**	**GGGATACAGTTGGGTTCAAACAC**
**TRAP**	**Sense**	**CATGACCACCTTGGCAATGTCTC**
	**Antisense**	**CTGTGGGATCTTGAAGTGCAGG**
**ITGB1**	**Sense**	**GGATTCTCCAGAAGGTGGTTTCG**
	**Antisense**	**TGCCACCAAGTTTCCCATCTCC**
**ITGB2**	**Sense**	**AGTCACCTACGACTCCTTCTGC**
	**Antisense**	**CAAACGACTGCTCCTGGATGCA**
**ITGB3**	**Sense**	**CATGGATTCCAGCAATGTCCTCC**
	**Antisense**	**TTGAGGCAGGTGGCATTGAAGG**
**IFNG**	**Sense**	**GAGTGTGGAGACCATCAAGGAAG**
	**Antisense**	**TGCTTTGCGTTGGACATTCAAGTC**
**MCSF**	**Sense**	**TGAGACACCTCTCCAGTTGCTG**
	**Antisense**	**GCAATCAGGCTTGGTCACCACA**
**TNF**	**Sense**	**CTCTTCTGCCTGCTGCACTTTG**
	**Antisense**	**ATGGGCTACAGGCTTGTCACTC**

The efficiency of the newly designed primers was confirmed by sequencing the conventional PCR products.

### 5.14 RNA Sequencing

Total RNA was extracted from PDLCs subjected to mechanical loading with or without SMFs stimulation, with three replicate samples prepared for each group. Subsequently, the samples were sent to Novogene Co., Ltd. for subsequent sequencing. Ultimately, raw reads exceeding 50 base pairs in length and meeting quality control standards were selected for further analysis. Fragments Per Kilobase of transcript per Million mapped reads (FPKM) values were applied for gene normalization and quantification. Gene Ontology (GO) and Kyoto Encyclopedia of Genes and Genomes (KEGG) analyses were employed to elucidate the biological functions of differentially expressed genes between the two groups.

### 5.15 Statistical Analysis

All measurements were conducted a minimum of 3 times, with results expressed as the average values along with their standard deviations. Shapiro‐Wilk test was employed to verify the normality of the data distribution. Differences between groups were assessed with Student's t tests or 1‐way analyses of variance

 unless otherwise stated. All statistical analyses were performed with Prism 9.5 (GraphPad Inc.) or SPSS 17.0 (IBM). P < 0.05 was considered statistically significant.

## Author Contributions

Yiling Lou: Contributed to design, data acquisition, analysis and interpretation, drafted and critically revised the manuscript. Jiwei Sun: Contributed to conception, data interpretation, drafted and critically revised the manuscript. Peng Chen: Contributed to design, data acquisition and analysis, critically revised the manuscript. Caihong Mao: Contributed to design, data analysis, drafted and critically revised the manuscript. Junyuan Zhang: Contributed to design, critically revised the manuscript. Haoqi Lei: Contributed to data acquisition and analysis, critically revised the manuscript. Xiaoqing Han: Contributed to data acquisition, critically revised the manuscript. Yaxuan Zhang: Contributed to data analysis, critically revised the manuscript. Yuheng Jiang: Contributed to data analysis, critically revised the manuscript. Yulin Jia: Contributed to conception, critically revised the manuscript. Bin Su: Contributed to design, critically revised the manuscript. Lili Chen: Contributed to conception and design, critically revised the manuscript. All authors gave their final approval and agree to be accountable for all aspects of the work.

## Funding

This project was supported in part by grants from the National Key R&D Program of China (2021YFC2400400/04, to LC), the Open Project for State Key Laboratory of Oral Diseases (SKLOD2024OF05, to JS), the National Natural Science Foundation of China (82401191, to JS).

## Conflicts of Interest

The authors declared no conflicts of interest.

## Ethical Approval

The research protocol was established, according to the ethical guidelines of the Helsinki Declaration, complied with the STROBE protocol and was approved by the Human Ethics Committee of Union Hospital, Tongji Medical College, Huazhong University of Science and Technology ([2024], 0334‐01). All animal studies were conducted with approval from the Institutional Animal Care and Use Committee, Huazhong University of Science and Technology ([2024] IACUC Number: 4153) and conformed to the updated ARRIVE 2.0 guidelines.

## Supporting information




**Supporting File**: advs77447‐sup‐0001‐SuppMat.docx.

## Data Availability

The datasets used and/or analysed during the current study available from the corresponding author on reasonable request.

## References

[1] G. Lombardo , F. Vena , P. Negri , et al., “Worldwide Prevalence of Malocclusion in the Different Stages of Dentition: A Systematic Review and Meta‐Analysis,” European Journal of Paediatric Dentistry 21, no. 2 (2020): 115–123, 10.23804/ejpd.2020.21.02.05. 32567942

[2] L. D'Onofrio , “Oral Dysfunction as a Cause of Malocclusion,” Orthodontics & Craniofacial Research 22, no. S1 (2019): 43–48, 10.1111/ocr.12277. PMC6851783 31074141

[3] L. Borsa , D. Estève , C. Charavet , and L. Lupi , “Malocclusions and Oral Dysfunctions: A Comprehensive Epidemiological Study on 359 Schoolchildren in France,” Clinical and Experimental Dental Research 9, no. 2 (2023): 332–340, 10.1002/cre2.719. PMC10098281 36934443

[4] Y. A. Yassir , G. T. McIntyre , and D. R. Bearn , “Orthodontic Treatment and Root Resorption: An Overview of Systematic Reviews,” European Journal of Orthodontics 43, no. 4 (2021): 442–456, 10.1093/ejo/cjaa058. 33215186

[5] A. S. Pinto , L. S. Alves , M. Maltz , C. Susin , and J. E. A. Zenkner , “Does the Duration of Fixed Orthodontic Treatment Affect Caries Activity among Adolescents and Young Adults?,” Caries Research 52, no. 6 (2018): 463–467, 10.1159/000488209. 29669345

[6] G. Nimeri , C. H. Kau , N. S. Abou‐Kheir , and R. Corona , “Acceleration of Tooth Movement During Orthodontic Treatment ‐ a Frontier in Orthodontics,” Progress in Orthodontics 14, no. 1 (2013): 42, 10.1186/2196-1042-14-42. PMC4384959 24326040

[7] I. Pouliezou , A. Xenou , K. Vavetsi , A. Mitsea , and I. Sifakakis , “Adverse Effects of Surgically Accelerated Orthodontic Techniques: A Systematic Review,” Children 9, no. 12 (2022): 1835, 10.3390/children9121835. PMC9777195 36553279

[8] M. A. Makrygiannakis , E. G. Kaklamanos , and A. E. Athanasiou , “Effects of Systemic Medication on Root Resorption Associated with Orthodontic Tooth Movement: A Systematic Review of Animal Studies,” European Journal of Orthodontics 41, no. 4 (2019): 346–359, 10.1093/ejo/cjy048. 29992228

[9] X. Duan , R. Zhang , H. Feng , et al., “A New Subtype of Artificial Cell‐Derived Vesicles from Dental Pulp Stem Cells with the Bioequivalence and Higher Acquisition Efficiency Compared to Extracellular Vesicles,” Journal of Extracellular Vesicles 13, no. 7 (2024): 12473, 10.1002/jev2.12473. PMC11223992 38965648

[10] B. Peng , Z. Li , Y. Cheng , H. Jiang , Q. Ye , and G. Han , “Dental Pulp Stem Cell‐Derived Intracellular Vesicles Prevent Orthodontic Relapse by Inhibiting PI3K/Akt/NF‐κB‐Mediated Osteoclast Activity,” Stem Cell Research & Therapy 16, no. 1 (2025): 22, 10.1186/s13287-025-04146-3. PMC11756174 39849611

[11] U. E. Ewii , A. A. Attama , E. O. Olorunsola , et al., “Nanoparticles for Drug Delivery: Insight into In Vitro and In Vivo Drug Release from Nanomedicines,” Nano TransMed 4 (2025): 100083, 10.1016/j.ntm.2025.100083.

[12] Y. Li , Q. Zhan , M. Bao , J. Yi , and Y. Li , “Biomechanical and Biological Responses of Periodontium in Orthodontic Tooth Movement: Up‐Date in a New Decade,” International Journal of Oral Science 13, no. 1 (2021): 20, 10.1038/s41368-021-00125-5. PMC8239047 34183652

[13] M. M. Weivoda and E. W. Bradley , “Macrophages and Bone Remodeling,” Journal of Bone and Mineral Research 38, no. 3 (2023): 359–369, 10.1002/jbmr.4773. PMC10023335 36651575

[14] S. Pongtiwattanakul , C. Leethanakul , O. Rattanaporn , P. Thammanichanon , and S. Tannukit , “Effect of Compressive Force Combined with Vibration on CCL2 and CCL5 in Human Periodontal Ligament Cells,” Journal of Oral Biology and Craniofacial Research 14, no. 5 (2024): 626–630, 10.1016/j.jobcr.2024.08.005. PMC11381868 39252795

[15] D. He , X. Kou , Q. Luo , et al., “Enhanced M1/M2 Macrophage Ratio Promotes Orthodontic Root Resorption,” Journal of Dental Research 94, no. 1 (2015): 129–139, 10.1177/0022034514553817. 25344334

[16] Y. Nakai , N. Praneetpong , W. Ono , and N. Ono , “Mechanisms of Osteoclastogenesis in Orthodontic Tooth Movement and Orthodontically Induced Tooth Root Resorption,” Journal of Bone Metabolism 30, no. 4 (2023): 297–310, 10.11005/jbm.2023.30.4.297. PMC10721376 38073263

[17] B. Alghamdi , H. H. Jeon , J. Ni , et al., “Osteoimmunology in Periodontitis and Orthodontic Tooth Movement,” Current Osteoporosis Reports 21, no. 2 (2023): 128–146, 10.1007/s11914-023-00774-x. PMC10696608 36862360

[18] N. F. Talic , “Adverse Effects of Orthodontic Treatment: A Clinical Perspective,” The Saudi Dental Journal 23, no. 2 (2011): 55–59, 10.1016/j.sdentj.2011.01.003. PMC3770235 24151415

[19] N. Luo , Y. Chen , L. Li , Y. Wu , H. Dai , and J. Zhou , “Multivariate Analysis of Alveolar Bone Dehiscence and Fenestration in Anterior Teeth after Orthodontic Treatment: A Retrospective Study,” Orthodontics & Craniofacial Research 27, no. 2 (2024): 287–296, 10.1111/ocr.12726. 37929647

[20] A. T. Semeano , F. A. Tofoli , J. C. Corrêa‐Velloso , et al., “Effects of Magnetite Nanoparticles and Static Magnetic Field on Neural Differentiation of Pluripotent Stem Cells,” Stem Cell Reviews and Reports 18, no. 4 (2022): 1337–1354, 10.1007/s12015-022-10332-0. 35325357

[21] W. Xie , C. Song , R. Guo , and X. Zhang , “Static Magnetic Fields in Regenerative Medicine,” APL Bioengineering 8, no. 1 (2024): 011503, 10.1063/5.0191803. PMC10939708 38486824

[22] R. Shimomura , S. Shibata , S. Koganemaru , et al., “Transcranial Static Magnetic Field Stimulation (tSMS) Can Induce Functional Recovery in Patients with Subacute Stroke,” Brain Stimulation 16, no. 3 (2023): 933–935, 10.1016/j.brs.2023.05.024. 37257816

[23] E.‐C. Kim , J. Park , G. Noh , et al., “Effects of Moderate Intensity Static Magnetic Fields on Osteoclastic Differentiation in Mouse Bone Marrow Cells,” Bioelectromagnetics 39, no. 5 (2018): 394–404, 10.1002/bem.22126. 29709064

[24] S. Wang , Y. Liu , C. Lou , et al., “Moderate Static Magnetic Field Promotes Fracture Healing and Regulates Iron Metabolism in Mice,” BioMedical Engineering OnLine 22, no. 1 (2023): 107, 10.1186/s12938-023-01170-3. PMC10647027 37968671

[25] Y. Shan , H. Han , J. Zhu , et al., “The Effects of Static Magnetic Field on Orthodontic Tooth Movement in Mice,” Bioelectromagnetics 42, no. 5 (2021): 398–406, 10.1002/bem.22346. 34033679

[26] K. Zhang , W. Ge , S. Luo , Z. Zhou , and Y. Liu , “Static Magnetic Field Promotes Proliferation, Migration, Differentiation, and AKT Activation of Periodontal Ligament Stem Cells,” Cells Tissues Organs 212, no. 4 (2022): 317–326, 10.1159/000524291. PMC10534995 35344952

[27] N. N. Alqaisi , R. A. Haddad , and H. M. Amasha , “Effectiveness of a Low‐Intensity Static Magnetic Field in Accelerating Upper Canine Retraction: a Randomized Controlled Clinical Trial,” BMC Oral Health 24, no. 1 (2024): 424, 10.1186/s12903-024-04212-x. PMC10998372 38582881

[28] B. B. Feyizoglu , B. H. Aysesek , S. Kaya , U. Baser , V. Olgac , and A. G. Isik , “Effects of Orthodontic Tooth Movement on Periodontal Tissues after Ligature‐Induced Periodontitis through the Mechanism of RANKL‐Induced Osteoclastogenesis: An Animal Study,” BMC Oral Health 25, no. 1 (2025): 1278, 10.1186/s12903-025-06627-6. PMC12312380 40739631

[29] S. Chaushu , Y. Klein , O. Mandelboim , Y. Barenholz , and O. Fleissig , “Immune Changes Induced by Orthodontic Forces: A Critical Review,” Journal of Dental Research 101, no. 1 (2022): 11–20, 10.1177/00220345211016285. 34105404

[30] H. Huang , R. Yang , and Y. Zhou , “Mechanobiology of Periodontal Ligament Stem Cells in Orthodontic Tooth Movement,” Stem Cells International 2018 (2018): 1–7, 10.1155/2018/6531216. PMC6166363 30305820

[31] D. H. J. Ng , J. D. Humphries , A. Byron , A. Millon‐Frémillon , and M. J. Humphries , “Microtubule‐Dependent Modulation of Adhesion Complex Composition,” PLoS ONE 9, no. 12 (2014): 115213, 10.1371/journal.pone.0115213. PMC4272306 25526367

[32] J. Aureille , S. S. Prabhu , S. F. Barnett , et al., “Focal Adhesions Are Controlled by Microtubules through Local Contractility Regulation,” The EMBO Journal 43, no. 13 (2024): 2715–2732, 10.1038/s44318-024-00114-4. PMC11217342 38769437

[33] N. B. M. Rafiq , Y. Nishimura , S. V. Plotnikov , et al., “A Mechano‐Signalling Network Linking Microtubules, Myosin IIA Filaments and Integrin‐Based Adhesions,” Nature Materials 18, no. 6 (2019): 638–649, 10.1038/s41563-019-0371-y. 31114072

[34] R. Luo , J. Dai , J. Zhang , and Z. Li , “Accelerated Skin Wound Healing by Electrical Stimulation,” Advanced Healthcare Materials 10, no. 16 (2021): 2100557, 10.1002/adhm.202100557. 33945225

[35] Z. Wan , P. Zhang , L. Lv , and Y. Zhou , “NIR Light‐Assisted Phototherapies for Bone‐Related Diseases and Bone Tissue Regeneration: A Systematic Review,” Theranostics 10, no. 25 (2020): 11837–11861, 10.7150/thno.49784. PMC7546009 33052249

[36] L. Caliogna , M. Medetti , V. Bina , et al., “Pulsed Electromagnetic Fields in Bone Healing: Molecular Pathways and Clinical Applications,” International Journal of Molecular Sciences 22, no. 14 (2021): 7403, 10.3390/ijms22147403. PMC8303968 34299021

[37] J. Yang , Y. Feng , Q. Li , and Y. Zeng , “Evidence of the Static Magnetic Field Effects on Bone‐Related Diseases and Bone Cells,” Progress in Biophysics and Molecular Biology 177 (2023): 168–180, 10.1016/j.pbiomolbio.2022.11.006. 36462638

[38] Y. Liu , Y. Guo , J. M. Valles , and J. X. Tang , “Microtubule Bundling and Nested Buckling Drive Stripe Formation in Polymerizing Tubulin Solutions,” Proceedings of the National Academy of Sciences 103, no. 28 (2006): 10654–10659, 10.1073/pnas.0510381103. PMC1502287 16818889

[39] S. Wang , J. Chen , H. Wang , et al., “Different Orientations Moderate Static Magnetic Fields Prevent Bone Loss and Improve the Mechanical Properties in Ovariectomized Mice,” Bioelectromagnetics 46, no. 8 (2025): 70037, 10.1002/bem.70037. 41424392

[40] L. Bondemark , “Orthodontic Magnets. A Study of Force and Field Pattern, Biocompatibility and Clinical Effects,” Swedish Dental Journal. Supplement 99 (1994): 1–148. 7801229

[41] T. Nakano , H. Hotokezaka , M. Hashimoto , et al., “Effects of Different Types of Tooth Movement and Force Magnitudes on the Amount of Tooth Movement and Root Resorption in Rats,” The Angle Orthodontist 84, no. 6 (2014): 1079–1085, 10.2319/121913-929.1. PMC8638510 24754797

[42] K. Kraiwattanapong and B. Samruajbenjakun , “Effects of Different Force Magnitudes on Corticotomy‐Assisted Orthodontic Tooth Movement in Rats,” The Angle Orthodontist 88, no. 5 (2018): 632–637, 10.2319/103117-736.1. PMC8183137 29714068

[43] T. Kohno , Y. Matsumoto , Z. Kanno , H. Warita , and K. Soma , “Experimental Tooth Movement under Light Orthodontic Forces: Rates of Tooth Movement and Changes of the Periodontium,” Journal of Orthodontics 29, no. 2 (2002): 129–136, 10.1093/ortho/29.2.129. 12114463

[44] M. Zou , C. Li , and Z. Zheng , “Remote Corticotomy Accelerates Orthodontic Tooth Movement in a Rat Model,” BioMed Research International 2019 (2019): 1–9, 10.1155/2019/4934128. PMC6601503 31317031

