# Supplementary material for: Arrayed Magnetic Accelerator Synergistically Enhances Orthodontic Tooth Movement via Microtubule Assembly‐Mediated Periodontal Remodeling
Source: Adv Sci (Weinh). 2026 Oct 4:e77447. Online ahead of print. doi: 10.1002/advs.77447 (PMC13634475; doi:10.1002/advs.77447)
Supplement: Supplementary file 1 [file ADVS-9999-e77447-s001.docx]

**Supplementary Materials**

**Supplementary Fig. 1** (a) The force value of the coil spring was accurately determined using a digital dynamometer. (b)(c) Hematoxylin and eosin (H&E) staining images of of liver and kidney sections proved the biosafety of the experiment in rats.

**Supplementary Fig. 2** (a)trabecular spacing (Tb. Sp), trabecular thickness between the roots of the maxillary first molar. Mean ± SD. n = 4. *P < 0.05, **P < 0.01. (b) tartrate-resistant acid phosphatase (TRAP) staining images of 28 days. R: root, PDL: periodontal ligament, AB: alveolar bone. Scale bar: 100 μm.(c) Statistics of numbers of TRAP- positive cells per mm of alveolar bone surface. Mean ± SD. n = 4. *P < 0.05, **P < 0.01. (d) Representative images of immunohistochemical staining of 28 days for IFN-γ in the periodontal ligament. Scale bar: 20 μm (e) Percentage of IFN-γ positive cells per field in the periodontal ligament of the first molar. Mean ± SD. n = 4. **P* < 0.05, ****P* < 0.001. (f) Representative immunofluorescence images of CD68-positive (green) and iNOS-positive (red) M1-like macrophages (merged yellow) of 28 days. Scale bar: 25 μm. (g)Percentage of M1-like macrophages per field. Mean ± SD. n = 4. *P < 0.05. (h) Percentage of CD68-positive cells per field in the periodontal ligament of the first molar. Mean ± SD. n = 4. **P < 0.01.

**Supplementary Fig. 3** (a) PDLCs were implanted in six-well plates and then placed on a custom device to apply SMFs. (b) The bionic pressure cell culture apparatus was used to apply vertical pressure (2.5mmhg) to PDLCs. (c) qRT-PCR results of *CTSK* expression in monocytes after 24 h of co-culture. Mean ± SD. *n* = 3. ***P* < 0.01.

**Supplementary Fig. 4** (a) Volcano map of differentially expressed genes (DEGs) in Force group vs Force + SMFs group.

**Supplementary Fig. 5** (a) Relative FPKM values of *ITGB5, CCL17* and *IL23A* from RNA-seq data. Mean ± SD. *n* = 3. **P* < 0.05, ***P* < 0.01, *****P* < 0.0001.
